# All-in-One Sustainable Thread Biosensor for Chemiluminescence Smartphone Detection of Lactate in Sweat

**DOI:** 10.3390/bios15080530

**Published:** 2025-08-13

**Authors:** Emanuela Maiorano, Maria Maddalena Calabretta, Eugenio Lunedei, Elisa Michelini

**Affiliations:** 1 Department of Chemistry “Giacomo Ciamician”, University of Bologna, Via P. Gobetti 85, 40129 Bologna, Italy; emanuela.maiorano3@unibo.it (E.M.); maria.calabretta2@unibo.it (M.M.C.); 2 Institute of Nanostructured Materials (ISMN)-National Research Council (CNR), Via P. Gobetti 101, 40129 Bologna, Italy; eugenio.lunedei@cnr.it; 3 IRCCS Azienda Ospedaliero-Universitaria di Bologna, 40138 Bologna, Italy

**Keywords:** thread-based biosensor, lactate, chemiluminescence, smartphone, sweat

## Abstract

Thanks to their low-cost, portability, and sustainability, microfluidic thread-based analytical devices (μTADs) are emerging as an attractive analytical platform for wearable biosensing. While several μTADs, mainly based on colorimetric and electrochemical detection methods, have been developed, achieving the needed sensitivity and accuracy for these biosensors continues to present a significant challenge. Prompted by this need we investigated for the first time the implementation of chemiluminescence (CL) as a detection technique for μTADs. Exploiting the lactate oxidase-catalyzed reaction coupled with the enhanced luminol/H_2_O_2_/horseradish peroxidase CL system, we developed a cotton-thread-based chemiluminescent device enabling the detection of lactate with a limit of detection of 0.25 mM in a 2 µL volume of artificial sweat at pH 6.5 within 3 min. The use of recycled grape skin as support made the device sustainable, while the smartphone detection allowed a simple and quantitative readout for the end-user. Using a smartphone as a detector, the analytical performance was evaluated in different conditions and in the presence of potential interferents, showing suitability for monitoring lactate levels in physiological conditions, such as for monitoring anaerobic thresholds in endurance training.

## 1. Introduction

Microfluidic-based devices are receiving significant attention for their advantageous properties in terms of portability, low-cost, and sustainability as well as in resource-limited settings. Microfluidic paper-based analytical devices have been widely developed for point-of-care (POC) diagnostics, environmental monitoring, and food safety control [1,2]. Traditionally, these devices have been fabricated using wax printing, a simple and cost-effective process involving the printing of patterns on paper, followed by heating of the wax for the formation of hydrophobic barriers. However, wax printers are no longer commercially available [3], prompting the need for alternative fabrication methods. One of these is represented by laser cutting, which offers faster processing times and higher precision than wax printing and it does not require heating steps [4]. Additionally, microfluidic thread-based analytical devices (μTADs) are emerging as a potential tools for low-cost biosensing based on the use of thread supports, which can be easily modified by chemical functionalization or immobilization of biomolecules without complex pre-treatments or special equipment [4,5,6]. Flexible designs of μTADs can be achieved by sewing, knitting, weaving, or twisting, offering the possibility to work with reduced sample volumes [7], making μTADs sustainable analytical tools in accordance with the principles of green chemistry.

Several μTADs have been reported since the pioneering work of Shen’s group [8], mostly relying on electrochemical detection, having the potential to provide a novel generation of electronic textile (e-textile) biosensors able to continuously monitor biomarkers at a physiologically relevant level [9,10,11]. Also, colorimetric μTADs have been reported, especially for monitoring biomarkers present at medium/high concentrations in sweat [12], with the potential for creating textile and wearable sensors that do not require batteries or any energy for working [13,14]. Sweat is a clinical matrix that has high potential for wearable diagnostics since it does not require invasive or painful procedures for sampling and contains a high number of clinical biomarkers [15,16], including glucose and lactate [17]. Lactate monitoring in sweat is of particular interest since it provides information about individual metabolic status during exercise, thus enabling non-invasive monitoring of athletic performance [18]. Another advantage of colorimetric μTADs is their possibility of being interfaced with smartphone detection. Smartphone-enabled analysis offers real-time, convenient data interpretation without the need for specialized equipment [19] and, in combination with artificial intelligence, provides enhanced user accessibility [20].

In this context, chemical luminescence, which has been widely combined with smartphone detection [21,22], could be highly advantageous, combining high sensitivity with instrument-free analysis. Both bioluminescence (BL) and chemiluminescence (CL) reactions produce photons as a result of chemical reactions, thus being advantageous in terms of high sensitivity (zero background and high quantum yield), high specificity (almost zero background), speed of reaction, and multiplexing capability [23,24]. 

Shimazu et al. provided the first proof-of-principle of implementing BL in μTADs [25]. Cotton threads were used as a platform for embedding BL-based sensing with the enzyme (luciferase) and its substrate (luciferin) on intertwisted threads. This method was successfully applied for antibody detection in very low amounts of whole blood (5 μL). CL detection has been widely used in paper-based devices, and a few studies suggest its suitability for cloth-based biosensors [26]; however, it has not yet been investigated in thread-based biosensors. To the best of our knowledge, we report for the first time the development of a CL thread-based biosensor for monitoring lactate in sweat. The biosensor integrates all reagents (enzymes and substrates) for the measurement, thus providing a user-friendly “add and measure” procedure, which simply requires 2 µL of sample for the analysis and a picture taken with a smartphone. The CL biosensor is composed of a microfluidic thread device functionalized with lactate oxidase (LOx) and horseradish peroxidase (HRP) enzymes, and luminol sewn on a recycled grape skin support. Lactate detection is measured through a coupled enzymatic reaction that exploits LOx and HRP enzymes, leading to light emission. The biosensor showed good analytical performance in artificial sweat at different pH (5.5, 6.5, and 8.0) without being affected from potential interferents present in sweat or other clinical matrices.

## 2. Materials and Methods

### 2.1. Materials and Instruments

Horseradish peroxidase Type VI-A (HRP), L-lactate oxidase (LOx) from *Aerococcus viridans* sp., L-histidine monohydrochloride monohydrate, sodium chloride, disodium hydrogen orthophosphate dodecahydrate, α-D-glucose, uric acid (UA) sodium salt, urea, bovine serum albumin (BSA), and cholesterol were supplied by Sigma Aldrich (St. Louis, MO, USA). Ascorbic acid (AA) was supplied by VWR International BV (Geldenaaksebaan, Belgium). Stock solutions of the enzymes (HRP: 301 U mg^−1^ protein in 20 mM Tris-HCl buffer pH 7.5; LOx: 46 U mg^−1^ protein in 20 mM Tris-HCl buffer pH 7.5) were prepared and stored at −20 °C. The Luminol SuperSignal™ ELISA Femto Maximum Sensitivity Substrate was from Thermo Scientific (Waltham, MA, USA). L-lactate sodium salt was supplied from Abmole Bioscience Inc. (Houston, TX, USA). The white cotton thread (N50) used for fabricating the device was purchased from Tomaselli Antonio company (Brindisi, Italy). The threads were washed using ultrapure water (18.2 MΩ cm) from a PURELAB flex water purification system (ELGA, Veolia Water, Marlow, UK). The threads were manually intertwisted, then sewn onto a natural skin layer obtained from grape waste provided by Krocette company (F.G.P.A. GROUP S.R.L.S., Roma, Italy).

For the acquisition of chemiluminescence signals, a OnePlus 6T smartphone (OnePlus, Shenzhen, China), equipped with an integrated camera (1/2.8″ 16 MP Sony IMX 398 sensor, 1.12 μm pixel size and F1.7 aperture) was used.

### 2.2. Fabrication of the Thread-Based Analytical Device

To obtain the thread-based analytical device for CL-coupled enzymatic reactions, 40 cm of a white cotton thread (N50, Tomaselli Tessuti, Francavilla Fontana, Italy) was first cut and inserted into a needle to separate the two strands (20 cm each) with a V-like shape. The two sides were then fixed, washed with 20 µL of MQ water per side, and let dry for 10 min at room temperature (25 ± 2 °C). Then, the two thread sides were first functionalized with luminol (SuperSignal™ ELISA Femto Maximum Sensitivity Substrate) and HRP enzyme (0.006 U) for testing the luminol/H_2_O_2_/ HRP CL system and subsequently with luminol and HRP + LOx for the CL-coupled enzymatic reaction.

### 2.3. Horseradish Peroxidase (HRP)/Luminol/H_2_O_2_ Reaction

The thread was previously washed with 20 µL of MQ water per side and dried for 10 min. A 20 µL volume of commercial luminol (1 µL per cm) was poured on one side of the thread, while a volume of 20 µL of HRP (1 µL per cm) was absorbed on the same thread. After drying (30 min at 25 °C), the two arms of thread were manually intertwisted to bring the enzyme HRP and the substrate luminol in proximity. Then the twisted thread was manually sewn onto a piece of recycled grape skin (Krocette company, F.G.P.A. GROUP S.R.L.S., Roma, Italy), making a sewing pattern (1 cm × 1 cm) with X-like shape. CL measurements were performed in a black box after the addition of 2 μL of H_2_O_2_ (concentration range from 15.0 to 100.0 µM) dispensed on the center of X-shaped pattern. 

### 2.4. Optimization of the Lactate Oxidase (LOx) and Horseradish Peroxidase (HRP) Coupled Enzymatic Reaction

**Figure 1:**
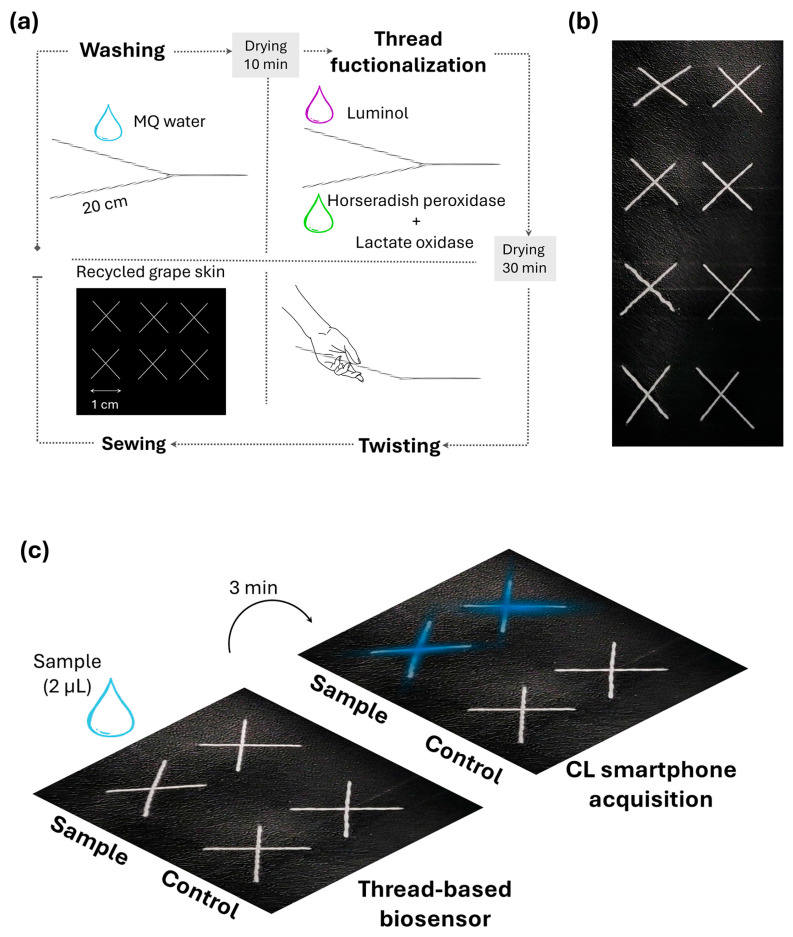
(**a**) Schematic illustration of the thread-based biosensor production: after washing and drying, HRP and LOx enzymes, and luminol substrate are separately adsorbed on two cotton threads, then intertwisted and sewn on recycled grape skin support. (**b**) Image of the thread-based biosensor sewn on recycled grape skin; (**c**) schematic analytical procedure for lactate detection with the thread-based biosensor.

After washing with 20 µL of MQ water per side and drying for 10 min, a 20 µL volume of commercial luminol (1 µL per cm) was poured on one side of the thread, while a volume of 20 µL of LOx and of HRP (ratio 1:1) was co-absorbed on the other side of the same thread (1 µL per cm). The enzyme amounts immobilized were as follows: LOx 1.0 U, HRP 0.006 U. After drying for 30 min at 25° C, the two parts of each thread were manually intertwisted to bring the enzymes, HRP and LOx, and the substrate luminol in proximity. The twisted sides of the thread were manually sewn onto a piece of black recycled grape skin kindly provided by Krocette (F.G.P.A. GROUP S.R.L.S., Roma, Italy), making a sewing pattern (1 cm × 1 cm) with X-like shape (Figure 1a,b). Recycled grape skin support (thickness 1.1 ± 10%) was composed of 80% Eco-Composite™ (comprising vegetal, renewable, and recycled raw materials), 20% water-based polyurethane with a backing part composed of recycled polyester, and a coating composed of 55% vegetal resins and grape and 45% water-based polyurethane.

CL measurements were performed in a black box after the addition of 2 μL of lactate (concentration range from 0.1 to 10.0 mM in 20.0 mM Tris-HCl buffer pH 7.5) dispensed on the center of X-shaped sewing pattern.

### 2.5. Analytical Assay Procedure for Lactate Monitoring in Artificial Sweat

The analytical procedure involves (i) addition of 2 μL of sample in the center of X-shaped sewing pattern; (ii) placement of the thread-based biosensor into a black box and 3 min incubation time at room temperature (25 ± 2 °C); (iii) CL acquisition (Figure 1c).

Different lactate solutions (concentrations range from 0.1 to 5.0 mM) were prepared in artificial sweat at different pH (5.5, 6.5, and 8.0), according to the International Standard Organization (ISO105-E04-2008E) and the British Standard (BS EN1811-1999) (pH 5.5: 0.05% *w*/*v* of L-histidine monohydrochloride monohydrate, 0.50% *w*/*v* of NaCl, and 0.22% *w*/*v* of NaH_2_PO_4_·2H_2_O; pH 6.5: 1.08% *w*/*v* of NaCl, 0.13%*w*/*v* of urea; pH 8.0: 0.05% *w*/*v* of L-histidine monohydrochloride monohydrate, 0.50% *w*/*v* of NaCl, and 0.50% *w*/*v* of Na_2_HPO_4_·12H_2_O) and tested with the thread-based biosensor.

### 2.6. Chemiluminescence Signal Acquisition and Data Analysis

CL measurements were performed in duplicate, and experiments were repeated at least three times. The background CL signal was measured by replacing the sample solution with buffer solution (buffer carbonate pH 10.0 luminol/H_2_O_2_/horseradish peroxidase (HRP) CL system, Tris-HCl buffer pH 7.5, sweat pH 5.5, pH 6.5, pH 8.0, for the coupled-reaction system that exploited LOx and HRP). The CL signals were acquired using a OnePlus 6T smartphone’s built-in camera, with a 30 s exposure time and ISO 3200. The focus was fixed on the sewn thread throughout the experimental procedure for consistent image sharpness. Measurements were performed in dark box to avoid ambient light interference. Images were captured in JPEG format using the device’s native camera application with default color profile settings. No cropping or digital magnification was applied to the images. Quantitative analysis of the CL images was performed with Image J software (v. 1.54g, National Institutes of Health, Bethesda, MD, USA), without correction for the gamma encoding (γ ≈ 2.2) automatically applied by the smartphone during JPEG processing. While this may introduce non-linearity in the concentration–response relationship [27], the reported detection limits remain valid under the consistent imaging conditions used. Future implementations could benefit from capturing images in RAW format or applying appropriate linearization corrections [27,28].

A circular region of interest (ROI) was chosen to define both the sample and control detection area as well as dark area of each image for the background subtraction. The CL signal was evaluated by integrating the CL image intensity over the ROI area using the “Measure” function, which calculates the “Mean Gray Value” for the selected region. The software automatically converts RGB color images to grayscale using the standard weighted formula (Gray = 0.299 × R + 0.587 × G + 0.114 × B). The corrected CL signal was therefore calculated as difference between the sample Mean Gray Value and the background Mean Gray Value. This method integrates signal across the entire visible spectrum through RGB-to-grayscale conversion, making it independent of the specific emission wavelength characteristics of different chemiluminescent systems. GraphPad Prism v. 8.3.0 (GraphPad Software, Inc., La Jolla, CA, USA) was used to plot the CL signal as a function of lactate concentration and for fitting data with dose–response (three-parameter) non-linear regression. The limit of detection (LOD) was calculated as the mean value of the blank (buffer carbonate pH 10.0, Tris HCl pH 7.5, sweat at different pH according to the system taken into consideration) minus three times the standard deviation of the control (buffer carbonate pH 10/Tris HCl pH 7.5/sweat at different pH). The limit of quantification (LOQ) was calculated as the mean value of the blank (buffer carbonate pH 10.0, Tris HCl pH 7.5, sweat at different pH according to the system taken into consideration) minus ten times the standard deviation of the control (buffer carbonate pH 10.0, Tris HCl pH 7.5, sweat at different pH according to the system taken into consideration).

### 2.7. Selectivity and Stability Studies

Selectivity studies were performed to evaluate the specificity of the thread-based biosensor in presence of potential interferents. Bovine serum albumin (BSA, 35 mg/mL in PBS 0.1 M), cholesterol (0.43 mM in 0.1 M NaOH), glucose (0.25 mM), ascorbic acid (0.1 mM), uric acid (0.1 mM in 0.1 M NaOH, 0.18 M NaCl), urea (21.7 mM) were tested using the same procedure described previously. Results were compared to those obtained with the thread-based biosensor with 1.0 mM lactate (in Tris-HCl 20.0 mM, pH 7.5). For stability studies, several thread-based biosensors were stored at +4 °C and tested for a 20-day period. The stability was evaluated by dispensing 2 μL of 1 mM lactate in Tris-HCl (20.0 mM, pH 7.5) on the center of X-shaped pattern at different time points. Each experiment was carried out at least in duplicate. CL signals obtained on day 0 were normalized as 100% of the signal.

## 3. Results and Discussion

### 3.1. Design and Fabrication of the CL Thread-Based Biosensor

To implement chemiluminescence in thread-based biosensing, we developed a biosensor based on cotton thread for monitoring lactate in sweat. To increase the sustainability of the approach, recycled grape skin was used as support to sew the cotton thread functionalized with enzymes and luminol.

The coupled enzymatic reaction catalyzed by LOx and HRP was exploited: in the first reaction catalyzed by LOx (Equation (1)), lactate is converted into pyruvate and H_2_O_2_; the subsequent reaction of H_2_O_2_ with luminol is catalyzed by HRP (Equation (2)), leading to emission of photons proportional to H_2_O_2_ concentration. Emitted light was measured with a smartphone without requiring specialized instrumentation.

(1) $$\mathrm{Lactate}+O_{2}\;\overset{LOx}{\to }\;\mathrm{Pyruvate}+H_{2}O_{2}$$

(2) $$H_{2}O_{2}+\mathrm{Luminol}\;\overset{HRP}{\to }\;\mathrm{Aminophthalate}+N_{2}+hv$$

Since this is the first time that chemiluminescence was implemented in a thread-based device, we preliminary investigated the emission properties of the luminol/H_2_O_2_/ HRP system on cotton thread to assess signal intensity and kinetics and perform a feasibility evaluation of this approach. A 2 μL volume of H_2_O_2_ (concentration range from 0.0 to 100 µM, buffer carbonate pH 10.0) was dispensed on the center of X-shaped pattern containing the luminol (SuperSignal™ ELISA Femto Maximum Sensitivity Substrate) and HRP (0.006 U). The CL reaction is triggered after hydrogen peroxide addition, which diffuses along the thread under the action of capillary forces, entering in contact with the luminol substrate and HRP. The spatial separation between the luminol and the HRP made by dispensing them on different sides of the thread prevents premature mixing and reaction of the reagents. CL emission was acquired for 4 min and analyzed with a third-order polynomial (cubic) equation. A maximum signal was obtained after 60-120 s from hydrogen peroxide addition (Figure S1); therefore, acquisitions were performed at 60 s, providing a detection limit for H_2_O_2_ of 25.00 µM. The natural properties of cotton thread allowed a controlled fluid flow with a wicking rate of 0.23 ± 0.04 cm/s and the possibility of handling a smaller sample volume (2 µL) in comparison to paper-based devices in a short time (5 min) [5,6,29]. The LOD obtained with our thread biosensor was comparable to a previously reported electrogenerated chemiluminescence (ECL) cloth biosensor relying on a luminol/H_2_O_2_-based ECL system that detected H_2_O_2_ with LOD of 0.024 mM [28]. Moreover, by attaching the threads to a support, there is no need to create hydrophobic barriers like those produced through wax printing, an approach that is currently limited due to the unavailability of wax printers.

### 3.2. Optimization of Thread-Based Device for Lactate Detection

After having identified the optimal conditions for HRP-catalyzed CL reaction, the coupled enzymatic reaction exploiting LOx and HRP was then optimized on cotton thread. Once the thread-based biosensor was sewn on recycled grape skin and previously functionalized with luminol on one side and HRP/LOx enzymes on the other side, a 2 μL volume of lactate was dispensed on the center of the X-shaped pattern. A concentration range of 0.1–10.0 mM was selected considering the physiological levels and the potential application of the biosensor to monitor sweat lactate during physical exercise [30]. An all-in-one thread biosensor was designed embedding all reagents required for the assay; the CL reaction was triggered after sample addition; sample diffused by capillarity along the thread, entering in contact with luminol and enzymes (HRP and LOx).

**Figure 2:**
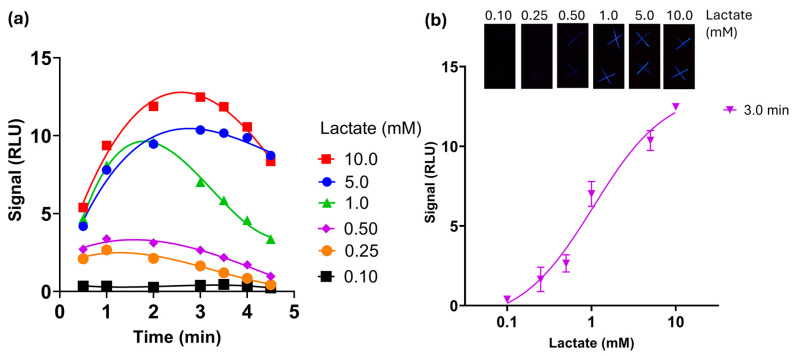
(**a**) Chemiluminescence emission kinetics obtained with different lactate concentrations (from 0.1 to 10.0 mM). Kinetic measurements were performed by acquiring CL signals with Oneplus 6 camera (ISO 3200, 30 s); (**b**) lactate calibration curve and CL images of thread-based biosensor taken 3 min after sample addition.

To define the optimal temporal window for CL measurements, pictures were acquired with the smartphone and analyzed as .jpeg pictures in ImageJ v. 1.53 k, summing the three channel values as part of the integration process. CL kinetic emissions were obtained after the addition of lactate (from 0.1 to 10.0 mM). As shown in Figure 2a, maximum emission intensities were obtained after 1–2 min from lactate addition at low concentrations (0.10, 0.25, 0.50 mM) and between 2 and 4 min for higher concentrations (5 and 10 mM). Lactate calibration curves were obtained at 3.0, 3.5, and 4.0 min (Figure 2 and Figure S2). CL intensities decreased proportionally with the decrease in lactate concentration, except at 4 min, when the variability between replicates increased (e.g., CV% for 1.0 mM lactate was 11% at 3 min vs. 22% obtained at 4 min).

According to the calibration curves, measurements were performed 3 min after sample addition. A limit of detection (LOD) of 0.15 mM was obtained, calculated as the lowest lactate concentration that produced a signal three standard deviations greater than the background signal (Figure 2b). Table S1 reports the LOD and LOQ obtained at the different time periods.

Considering the emission wavelength of luminol/HRP chemiluminescence (λ ≈ 431–445 nm), analysis of the blue channel only was also performed on ImageJ v. 1.53 k to improve signal-to-noise ratios. As shown in Figure S3, blue channel detection demonstrated higher signal-to-noise ratios across all concentrations tested. Despite enhanced signal intensities, both methods achieved comparable limits of detection (blue channel: 0.17 mM; grayscale: 0.15 mM), leading to adoption of the grayscale (RGB) approach for universal applicability across diverse luminescent systems. Results (Figure S3) showed enhanced signal intensities but comparable limits of detection between the two methods (0.17 mM for the blue channel), leading to the adoption of the grayscale (RGB) approach for its broad applicability across various types of emitters without requiring spectral optimization for each specific system.

Unlike other biosensors for sweat lactate analysis [31], the proposed CL cotton-thread biosensor is cost-effective, simple to manufacture, and does not require the creation of hydrophobic barriers. Moreover, the spatial separation between the CL substrate (luminol) and the CL enzymes (HRP and LOx), exploiting the natural flexibility of the cotton thread, prevented the premature mixing and reaction of the reagents. This helped with selecting the most suitable time window for CL measurement while reducing the volume and amount of reagents, in contrast to the most common paper-based devices, μPADs, which may require longer assay time and higher sample volumes (generally 10–30 µL) [32].

The thread-based device is highly competitive compared to other fiber material-based microfluidic devices including microfluidic cloth-based analytical devices (μCADs). For example, a chemiluminescence μCAD showed an LOD of 0.46 mM H_2_O_2_ [33], which is higher than the LOD here-reported (25.00 µM).

### 3.3. Detection of Lactate in Spiked Artificial Sweat and Recovery Studies

**Figure 3:**
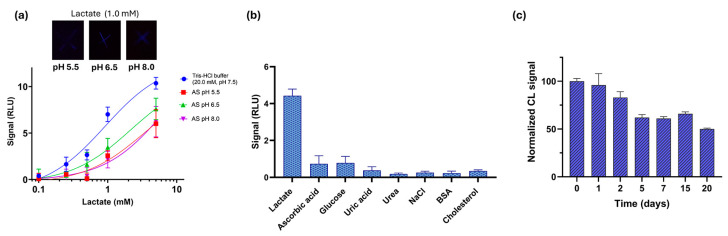
(**a**) Calibration curves for lactate (concentrations ranging from 0.01 mM to 5.0 mM) of the thread-based biosensor in Tris-HCl (pH 7.5) and artificial sweat at pH 5.5, 6.5, and 8.0 obtained with the optimized assay conditions. Smartphone pictures (3200 ISO, 30 s) were taken after 3 min incubation with 1.0 mM lactate at pH 5.5, pH 6.5, and pH 8.0. (**b**) Selectivity studies of the CL thread biosensor with different interferents present in human sweat and other clinical matrices. (**c**) Stability studies of the CL thread biosensor stored at +4 °C for 20 days (details in Section 2).

The pH of human sweat ranges from 4.0 to 7.0 in normal conditions [33,34], with an average pH of 6.3. The applicability of the thread-based CL biosensor for the analysis of lactate in artificial sweat at different pH was also evaluated. As shown in Figure 3a, lactate calibration curves (concentration range from 0.1 to 5.0 mM) were obtained in artificial sweat prepared at pH 5.5, 6.5, and 8.0 according to the International Standard Organization (ISO105-E04-2008E) and the British Standard (BS EN1811-1999) [35], obtaining LODs of 0.27, 0.24, and 0.16 mM, respectively. LOQs at pH 5.5, 6.5, and 8.0 were 0.40, 0.65, and 0.52, respectively. Higher variability between replicates was found at higher concentrations (5.0 mM) for samples at pH 5.5 and pH 8.0. These results confirm the possibility of using the thread biosensor to measure physiological lactate levels in human sweat [36,37]. The biosensor showed good analytical performance when compared to other recently reported biosensors for lactate detection in sweat, such as an electrochemical thread-based nano-biosensor developed by Zhao et al. [10] and an electrochemical µPAD [38], which showed an LOD of 3.61 mM and a linear range from 0.5 to 4 mM, respectively.

In addition, the degree of sustainability of the thread-based biosensor was assessed according to White Analytical Chemistry principles [39] and compared to other biosensors for lactate monitoring previously reported [40,41]. The sustainability (green score) and practicability (blue score) of the biosensor were very high, i.e., 97.5% and 95.9%, respectively, corroborating further developments of the biosensor for low-carbon-footprint point-of-care diagnostics (details in Supplementary Materials, Tables S2–S4).

### 3.4. Biosensor Interference and Stability Studies

Selectivity studies were performed to evaluate the specificity of the thread-based CL biosensor. The effect of potential interferents including compounds present in human sweat and other clinical matrices was evaluated. The assay was performed as previously described using 2 µL of BSA (35 mg/mL), cholesterol (0.43 mM), glucose (0.25 mM), ascorbic acid (0.1 mM), uric acid (0.1 mM), NaCl (0.18 M), urea (21.7 mM). These concentrations encompass and, in some cases, exceed physiologically relevant ranges found in human sweat, providing selectivity validation under both normal and extreme perspiration conditions [35,37,41]. The CL signals were compared to those obtained with 1.0 mM lactate. As shown in Figure 3b, all the tested compounds did not produce a significant CL signal, confirming the suitability of the biosensor for analyzing sweat in complex matrices.

Thread-based device stability was evaluated after storing the device at +4 °C. The analytical performance of the biosensor for the detection of lactate was assessed after 24 h, 2 days, 5 days, 7 days, 15 days, and 20 days of storage at 4 °C, acquiring the CL images and normalizing the CL signals to the values obtained for the freshly prepared biosensor (day 0). As shown in Figure 3c, storage of the biosensor at 4 °C resulted in a decrease in the CL signal of 4% on day 1, of 17% on day 2, and of 38, 39, and 34% on days 5, 6 and 15, respectively. Its activity halved on day 20. These values are consistent with a loss of enzyme activity due to the absence of preserving agents but still promising for real-life applications. Future activity will focus on the implementation of strategies to extend the shelf-life of the biosensor.

## 4. Conclusions

Microfluidic thread-based analytical devices present a promising platform for point-of-care applications and wearable sensing. Here we introduce for the first time the implementation of chemiluminescence (CL) biosensing on a cotton thread platform for lactate detection in sweat. By utilizing the lactate-oxidase-catalyzed reaction in combination with the enhanced luminol/H_2_O_2_/horseradish peroxidase CL system and smartphone detection, we developed a thread-based CL device capable of detecting lactate with a limit of detection of 0.25 mM in a 2 µL volume of artificial sweat at pH 6.5 and 3 min incubation time. Thanks to smartphone analysis the total turnaround time was less than 5 min, ensuring ease of use for the end-user. The analytical performance of the biosensor was assessed under various pH conditions and in the presence of potential interferents, yielding satisfactory results for lactate monitoring during physical activity. Additionally, the use of recycled grape skin as a biocompatible support made it more sustainable than previously reported approaches relying on 3D-printed devices or electrochemical biosensing. In the future this approach could be adapted to detect diagnostic biomarkers in sweat, e.g., chloride for cystic fibrosis, which are currently performed using a patch to collect sweat and standard analytical instrumentation.

## Data Availability

The data presented in this study are available on request from the first author.

## Supplementary Materials

The following supporting information can be downloaded at https://www.mdpi.com/article/10.3390/bios15080530/s1, Figure S1: Chemiluminescence emission kinetics obtained with different H_2_O_2_ concentrations; Figure S2: Lactate calibration curve of thread-based biosensor at different time after sample addition; Figure S3: Dose-response curves for lactate detection comparing blue channel and grayscale (RGB) analysis methods; Table S1: LOD and LOQ of Lactate in 20 mM Tris-HCl buffer pH 7.5 for the different time periods; Table S2: Criteria assignment of blue principles scores; Table S3: Criteria assignment of green principles scores; Table S4: Comparison of the green and blue principles of the chemiluminescence thread biosensor for lactate with other biosensors and kits for lactate analysis.
