# Supplementary material for: All-in-One Sustainable Thread Biosensor for Chemiluminescence Smartphone Detection of Lactate in Sweat
Source: Biosensors (Basel). 2025 Aug 13;15(8):530. doi: 10.3390/bios15080530 (PMC12384807; doi:10.3390/bios15080530)
Supplement: Supplementary file 1 [file biosensors-15-00530-s001.zip › biosensors-3780295-supplementary.pdf]

Supporting Materials

All-in-one sustainable thread biosensor for chemiluminescence smartphone detection of lactate in sweat

Emanuela Maiorano[a], Maria Maddalena Calabretta[a], Eugenio Lunedei[b] and Elisa Michelini[a,c*]

[a]*Department of Chemistry "Giacomo Ciamician", University of Bologna, Via P. Gobetti 85, 40129, Bologna, Italy*

[b]*Institute of Nanostructured Materials (ISMN) - National Research Council (CNR), Via P. Gobetti 101, 40129, Bologna, Italy*

[c]*IRCCS Azienda Ospedaliero-Universitaria di Bologna, 40138 Bologna, Italy*

*Corresponding author:

Prof. Elisa Michelini

University of Bologna

Dept. of Chemistry "Giacomo Ciamician"

Via P. Gobetti 85, 40129 Bologna, Italy

elisa.michelini8@unibo.it

Table of contents

EXPERIMENTAL SECTION 3

Figure S1: Chemiluminescence emission kinetics obtained with different H2O2 concentrations (from 15 to 100 μM). 3

Figure S2: Lactate calibration curve of thread-based biosensor taken (a) 3.5 min and (b) 4.0 min after sample addition. 3

Table S1: LOD and LOQ of Lactate in 20 mM Tris-HCl buffer pH 7.5 for the different time periods 3

Figure S3: Dose-response curves for lactate detection comparing blue channel and grayscale (RGB) analysis methods. 4

ASSESSMENT OF SUSTAINABILITY 4

Blue principles 5

Table S2: Criteria assignment of blue principles scores according to Nowak et al. [37] for the sustainability assessment of chemiluminescence thread-based biosensor. 5

Green principles 5

Table S3: Criteria assignment of green principles scores according to Nowak et al. [1] for the sustainability assessment of the chemiluminescence thread-biosensor for lactate. Pictograms of the 'Globally Harmonized System of Classification and Labelling of Chemicals" are considered. 6

Table S4. Comparison of the green and blue principles of the chemiluminescence thread-biosensor for lactate developed in this work, an electrochemical paper sensor, a chemiluminescence 3D printed sensor, and spectrophotometric kits used as preference laboratory method for lactate analysis. 6

References 7

EXPERIMENTAL SECTION

Figure S1: Chemiluminescence emission kinetics obtained with different H2O2 concentrations (from 15 to 100 μM).

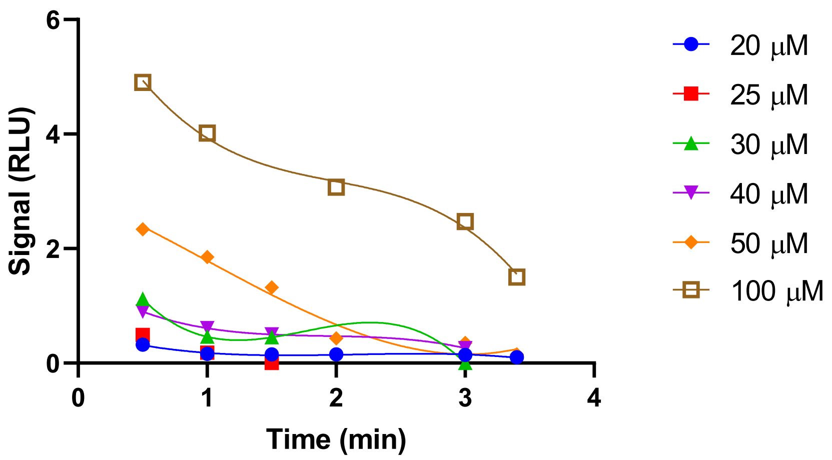

Figure S2: Lactate calibration curve of thread-based biosensor taken (a) 3.5 min and (b) 4.0 min after sample addition.

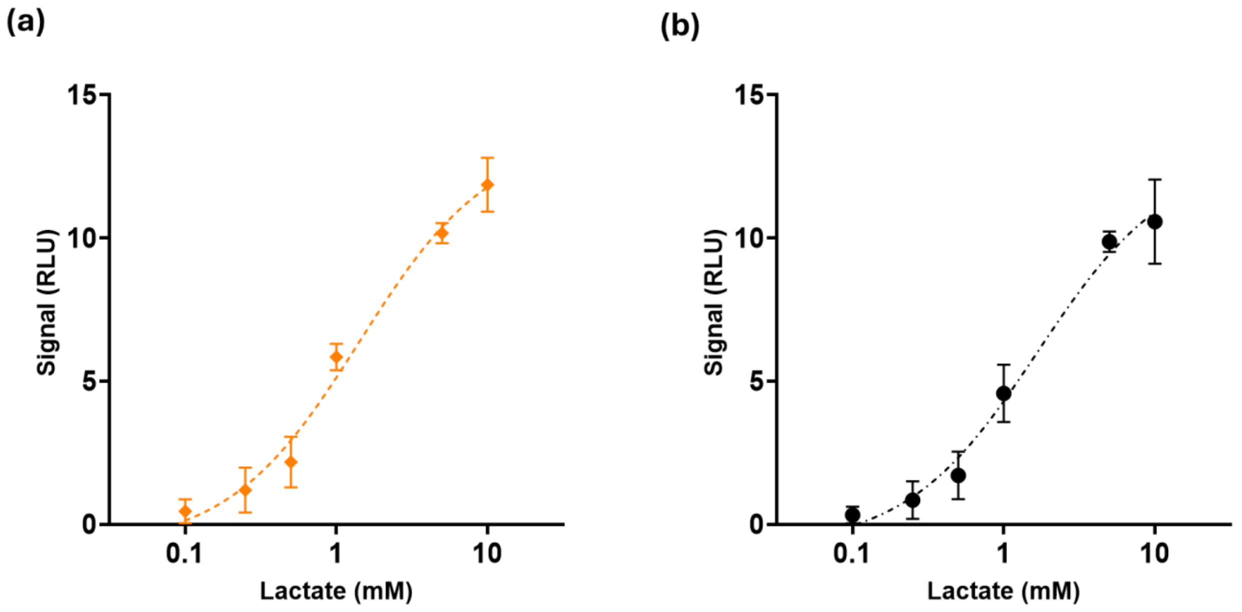

Table S1: LOD and LOQ of Lactate in 20 mM Tris-HCl buffer pH 7.5 for the different time periods

Time (minutes)	Limit of Detection, LOD (mM)	Limit of Quantification, LOQ (mM)
3	0.15	0.28
3.5	0.15	0.29
4	0.18	0.31

Figure S3: Dose-response curves for lactate detection comparing blue channel and grayscale (RGB) analysis methods.

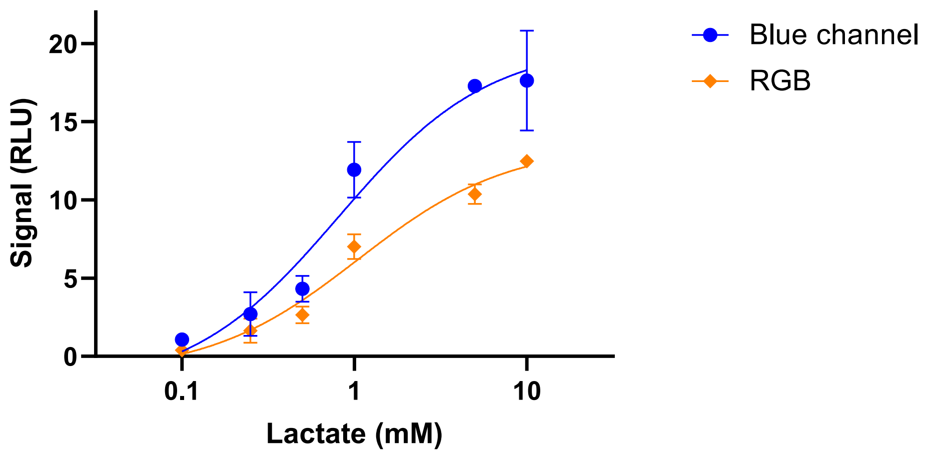

ASSESSMENT OF SUSTAINABILITY

The blue and green principles of the White Analytical Chemistry were addressed to assess the sustainability of the thread-based biosensor according to Nowak et al.[1]. The scores were tailored based on the key features of the blue and green principles applicable to a biosensor. The description of the selected parameters and scores is given in Table S2 for the blue principles and Table S3 for the green principles. The white principles, referring to analytical performance, were not considered for sustainability assessment.

Table S4 reports the scores for thread-based biosensor and a comparison with other sweat biosensors reported in the literature [1-3]. The low sample volumes (2 µL), used for the CL thread-based device, significantly reduced reagents consumption, contributing to sustainability and convenience in wearable or point-of-care applications, versus larger-volume methods like 3D-printed devices and spectrophotometry. Sensor's design and measurement protocols (e.g., enzyme immobilization, CL signals acquisition), were optimized for small sample volumes, reducing dilution and minimizing sample loss, thus mitigating risks of sampling errors. This is supported by existing research showing robust sensor responses even with micro- to nano-liter volumes [3].

Blue principles

B1. Cost-efficiency. Both reagents, materials, instrumentation and personnel cost are considered for the total analysis cost calculation.

B2. Time-efficiency. The time required for production of the thread biosensor and assay time (incubation with sample, detection and data analysis) are considered

B3. Requirements. The instrumentation, amount of sample, need for skilled personnel or specialized instrumentation are considered for biosensor fabrication and for carrying on the assay.

B4. Operational simplicity. All issues related to portability, automation and ease of use are considered in this score.

Table S2: Criteria assignment of blue principles scores according to Nowak et al. [37] for the sustainability assessment of chemiluminescence thread-based biosensor.

SCORE	B1-Cost efficiency	B2-Time efficiency (biosensor fabrication, analysis time)	B3-Requirements	B4-Operational simplicity
**0**	Very expensive	Within one week	Highly specific laboratory infrastructures / equipment	Requirements for sophisticated facilities and manufacturing equipment
**25**	Expensive	Within one day	Specific laboratory infrastructures / equipment	Requirements for basic manufacturing equipment, benchtop luminometers
**50**	Medium cost	Within half-day	Common laboratory infrastructures / equipment	Requirements for basic equipment, portable instrumentation
**75**	Low-cost	Within 3 hours	Minimal laboratory infrastructures / equipment	No need for equipment, need for benchtop luminometers
**100**	Very low-cost	Within less than two hours	No need for instrumentation	Full portability (No need for equipment and benchtop luminometers)

Green principles

G1: Toxicity of reagents. The safety data sheets for all the reagents, considering the quantity of pictograms, for every reagent was considered.

***G2:* Volume and n*umber of reagents and waste*.** The volumes of reagents were considered and compared to those reported in the cited literature (if available).

***G3. Energy and other media*.** Energy consumption required for handling fluidics or for measurement was considered.

***G4. Direct impacts*.** The impact on human beings connected to the used reagents was considered.

Table S3: Criteria assignment of green principles scores according to Nowak et al. [1] for the sustainability assessment of the chemiluminescence thread-biosensor for lactate. Pictograms of the 'Globally Harmonized System of Classification and Labelling of Chemicals" are considered.

SCORE	G1-Toxicity of reagents	G2-Waste produced	G3-Energy requirement	G4-Direct impacts
**0**	High toxicity, ≥ 10 pictograms (serious health hazard, acute toxicity)	Many reagents and high quantity of waste	High (more instruments> 1.5 kWh)	Dangerous activities
**25**	Significant health effects on living beings and the environment, 5 < pictograms < 10	Many reagents but discrete amount of waste	Significant (> 1.5 kWh)	Medium hazardous activities
**50**	Possible health effects on living beings and the environment; 2 < pictograms ≤ 5	Few reagents and discrete amount of waste	Medium (0.1 - 1.5 kWh)	Low hazardous activity
**75**	Low health effects on living beings and the environment (aqueous reagents); 1 < pictograms ≤ 2	Very few reagents and low amount of waste	Low (< 0.1 kWh)	Very low hazardous activity
**100**	Very low health effects on living beings and the environment (aqueous, non toxic and biodegradable reagents); 0 < pictograms ≤ 1	Minimal number of reagents and waste	None or very low	No hazardous activity

Table S4. Comparison of the green and blue principles of the chemiluminescence thread-biosensor for lactate developed in this work, an electrochemical paper sensor, a chemiluminescence 3D printed sensor, and spectrophotometric kits used as preference laboratory method for lactate analysis.

Configuration of lactate biosensor/method	Biosensor fabrication and use (technologies, material, re-usability,instrumentation)	Reagents a/Volumes	*Green* score (%)	*Blue* score (%)	Ref.
Thread biosensor with chemiluminescence smartphone detection	Cotton thread, recycled grape skin, smartphone	2 µL sample volume	97.5	95.9	This work

3D-printed device for smartphone-based chemiluminescence biosensor	ABS polymer, 3D printer, smartphone	15 µL sample volume	93.8	81.0	[2]
Electrochemical µPAD	3D bioprinting pen, laser cutter Whatman 41 filter paper	2 µL sample volume	93.8	94.2	[3]
Spectrophotometric assay for lactate quantification	Spectrophotometer	50 µL sample volume	64.2	62.1	Commercial kits (i.e., Labtests, Merck)

References

1. Nowak, P.M.; Wietecha-Posłuszny, R.; Pawliszyn, J. White Analytical Chemistry: An Approach to Reconcile the Principles of Green Analytical Chemistry and Functionality. *TrAC Trends in Analytical Chemistry* **2021**, *138*, 116223, doi:10.1016/j.trac.2021.116223.

2. Roda, A., Guardigli, M., Calabria, D., Calabretta, M.M., Cevenini, L., Michelini, E.. A 3D-printed device for a smartphone-based chemiluminescence biosensor for lactate in oral fluid and sweat. *Analyst.* **2014,** *24*, 6494–501. doi: 10.1039/c4an01612b

3. Berkheimer, Z.A.; Tahir, A.; Nordin, G.P.; Paixão, T.R.L.C.; Woolley, A.T.; do Nascimento, G.H.M.; de Araujo, W.R.; Pradela-Filho, L.A. Extruded Filament Electrodes for Lactate Biosensing in Continuous-Injection Paper-Based Microfluidic Devices. *Biosens Bioelectron* **2025**, *278*, 117390, doi:10.1016/j.bios.2025.117390.
